# Sharing in the life of the person with disability: A Ghanaian perspective

**DOI:** 10.4102/ajod.v4i1.185

**Published:** 2015-09-29

**Authors:** Frances E. Owusu-Ansah

**Affiliations:** 1 Department of Behavioural Sciences, Kwame Nkrumah University of Science and Technology, Ghana

## Abstract

This thought article was a hermeneutic inquiry into the experiences of informal caregivers of the elderly who are also physically disabled. The experiences of some Ghanaian informal caregivers were examined in three clinical cases and laced with the lived experiences of the author as an informal caregiver and clinician. Two processes were explored. The first relates to how a caregiver is changed through the experience of caregiving by examining the intrapersonal and interpersonal dynamics affecting caregiving. Secondly, the positive ‘shifts’ that occurred in therapy were explored. In the present Ghanaian society it appears that care for the elderly disabled is compounded by the rapid migration of many Ghanaians to ‘greener pastures’ in search of a brighter future, with consequent empty homesteads and fragmentation of the socio-cultural practices that hitherto buttressed informal care for the aged. In the absence of well-established professional care facilities, informal caregiving with its numerous challenges has become the norm for many. This article posited that caregiver self-care is the most important, and yet often forgotten, aspect of informal caregiving. When this is neglected, caregiver burnout is sure to occur, which results in poor physical, mental and emotional health for the caregiver. In this state caregivers may injure both themselves and the care recipients.

## Introduction

Ghana, like the rest of the world, is changing fast and losing some of its traditional systems and practices to modern ways of living with implications for the care of its disabled elderly. One specific societal change is the perception and definition of ‘family’. These days many think of family as mother, father and children - quite nuclear. This was not the case many years ago. Growing up, living with many members of the extended family was the norm. Everyone was ‘family’ and there was hardly any distinction between ‘real’ siblings and cousins, nephews, aunts and uncles. Even the architectural design of homes has changed. Back then homes were larger, circular in style, unwalled and spacious, suggestive of an ‘all are welcome’ attitude. Now, homes are generally smaller, walled and can only accommodate the parents and children, with one or two guest rooms. Extended family members are welcomed, but only for a while before they become a nuisance. It appears that the Ghanaian society is gradually losing its extended family culture, with implications for the provision of care for the elderly and disabled.

Another observation of a social change is the plethora of newly built homes that are sparsely inhabited and practically ‘empty nests’, mostly because the adult children of families have travelled abroad in search of greener pastures. Where there are remaining adult children in the country, or in the vicinity, they might not live with their parents because of their own responsibilities and busy lifestyle that make it difficult to live with or provide adequate care for elderly and disabled parents. The elderly disabled parent either lives alone or is primarily in the care of a house help. In a country like Ghana, where professional hospices are practically non-existent, hired hands or some family members are solicited as informal care providers. Provision of care in these situations is fraught with challenges, such as the quality of care provided, the caregiver's self-care, as well as the lack of support and understanding for the ways in which a caregiver is changed by the role and process of caregiving.

Ghana, like other predominantly collectivist African countries, prides itself in its community-oriented, interdependent way of life. It is a culture in which people's concept of *being human* is anchored in meaningful relationships. In the native languages, being ‘human’ (‘*nnipa*’) basically refers to the ability to relate well with others, to care about the well-being of another (Sarpong 1991, 2002). Therefore, within this social context, care for the elderly would not only be a cultural expectation, but would also be perceived as a human responsibility – a humane and noble task. In recent times, however, it is questionable if this is still true since many elderly and disabled parents are in the care of non-family hired hands, and some are neglected by family members. It appears that Ghanaian collectivist values are becoming a thing of the past, particularly in respect of the care of the elderly. It appears that the present Ghanaian society has a niche for non-family mercenary home care services for the aged disabled.

These reflections motivated the hermeneutic inquiry into the effects of informal caregiving as presented in the context of therapy. The method of phenomenology, as an inductive qualitative research, is rooted in Husserl and Heidegger's philosophical traditions (Reiners 2012). Heidegger's interpretive hermeneutic approach is used because it is better suited for this investigation. The everyday experiences of three informal caregivers of elderly disabled, who sought psychotherapy to support their role as caregivers, are described and interpreted to highlight the challenges within the Ghanaian cultural context. These women sought therapy at different times between March and December 2011. Their stories are recapitulated to delineate key challenges and issues reported. Their common experiences are described and interpreted to examine two things: firstly, how the caregiver is changed by the experience of caregiving and, secondly, what they found helpful in their self-care and recommended here for informal caregivers within the Ghanaian context.

According to Heidegger, it is impossible to negate own experiences about a phenomenon under study (Giorgi 2007). Therefore, the writer's experience as an informal caregiver and clinician are not bracketed. However, to ensure the validity of data and the emerged common themes obtained through content analysis, an independent reader was engaged to read the case reports for validation.

The main aim of this article is to bring more attention to ‘family caregivers’ (e.g. daughters) in Ghana who provide care for the disabled elderly and to highlight some of their peculiar challenges. These family caregivers could also be considered informal caregivers since they provide unpaid caregiving services to their disabled elderly. Primary informal caregivers are those who are solely and primarily responsible for the care recipient. A secondary informal caregiver is not solely responsible for the care recipient, but provides assistance to the primary caregiver (Goodhead & McDonald 2007).

## The concept and experience of caregiving

The Ghanaian culture values respect for the elderly. Generally, it is also a culture of interdependence within which children are the ‘social security’ of their parents and are expected to care for them in their elder years, particularly when they are infirm (Sarpong 1991). In this traditional context, care of the elderly would be considered a filial piety. This concept of caregiving, in the Ghanaian context, is rooted in the culture of the people. However, given the social demands in the life of the modern Ghanaian, it appears that some of these values are waning.

The experience of providing care for another, whether voluntary or compelled by life's circumstances, impacts both the caregiver and the care recipient. When it is voluntary, it can be an intrinsically fulfilling experience (Tang 2008), even in the midst of challenges (Akintola 2010). Several studies attest that a caregiver's quality of life is affected - positively, negatively or both. While some caregivers find fulfilment and greater resilience they did not know they possessed, others are negatively affected to the point of a burnout (Savage & Bailey 2004). The nature and extent of the impact is moderated by factors such as the caregiver's age, cultural background (Sörensen & Conwell 2011; Wallhagen & Yamamoto-Mitani 2006), attachment (Ainsworth 1989), intrapersonal resources or vulnerabilities, financial status, health or disability status, as well as the level of dependence of the care recipient (Savage & Bailey 2004). Generally, a review of the literature suggests that caregiving is a complex phenomenon that produces both psychological rewards as well as distress (Turner & Findlay 2012). The experience of caregiving, whether negative or positive, affects the caregiver's quality of life (Tang 2008) and ‘changes’ the caregiver (Savage & Bailey 2004).

Drawing from over eight years experience as an informal caregiver to an aged and visually impaired mother, the author knows that the experience of caregiving *does* change the caregiver in many ways. Among other things, it provides occasions for deep and personal introspection, reappraisal of own values, and opportunities for self-transcendence. It was during the time she took care of her visually impaired mother as a primary informal caregiver that she worked in her practice as a clinical psychologist with the women described in this article. Given her personal experience as a caregiver, she was delighted to have clients who were also primary informal caregivers to their elderly and disabled parents. She could relate, for example, to their experiences, sentiments, struggles and ‘resolutions’. Her caregiving experience enhanced her clinical skills and made her better able to understand and help them. Reflecting on her role as therapist in their ‘personal journeys’, and on her own experience as a caregiver, birthed this thought article. Therefore the experiences of these three women, presented below, were used to examine the intra- and interpersonal dynamics affecting caregiving in the Ghanaian context.

## Clinical cases^1^

### Case A

Amy is the third child of five - she has three sisters and a brother. She cares for her 82-year-old visually impaired and somewhat demented mother. She is the only one in Ghana as her four siblings live in Europe and North America. The death of their father, almost a decade earlier, saw the onset of her mother's health problems and eventual loss of sight. As a professional banker, Amy lives away from her mother and commutes almost daily for about an hour to visit and see to her needs before going home to her own family or to work. She is assisted by a hired hand, a young adult. Amy often wondered why she had accepted this role in the first place because, like her other siblings, she could have chosen ‘not to come back to Ghana’ after her studies abroad. Since her mother's memory is gradually deteriorating, her mother cannot remember Amy's visits and bitterly complains when she has not seen her for even a couple of days. Amy was deeply frustrated and angry with her siblings, who were reportedly ‘going on with their lives’ but ‘frequently phoned to give all kinds of instructions’ on how she should care for their mother. She sought therapy to deal with the pain and frustrations she experienced as her mother's caregiver. Her stated goals for therapy included how to deal with the depression, pain, frustrations, and ‘the helplessness of watching my mother deteriorate’, plus ‘other emotions I cannot even sort out’, she said.

### Case B

Evelyn is a 47-year-old woman who lives with her 71-year-old mother, who has diabetes and is hypertensive. Complications from the diabetes resulted in the amputation of one leg at the knee and is gradually causing visual impairment and incontinence. Evelyn is the sole caregiver because she is an only child and her own children are too young to give her meaningful assistance. By nature gregarious and extroverted, Evelyn looks forward to her daily quiet time with as little external input as possible, especially after a long day. But caring for a visually impaired mother who relies heavily on audio input to orient and to feel connected with the outside world requires that the radio in her home is constantly blurring. Even though she sincerely dislikes the constant ‘noise’ from the radio, she leaves it on ‘for mother's sake’ and ‘I put up with it’. She came to therapy emotionally and physically exhausted and consumed by the role of providing care. She wanted to ‘find an outlet’ and ‘someone to talk to’.

### Case C

Mercy is a 28-year-old mother of a 10-year-old boy who is mentally challenged with an expressive speech disorder. She is the sole caregiver of her son because ‘my husband blamed me for Kofi's problems and divorced me when he was barely two years old’. After the divorce she moved home to live with her parents. This placed her as an informal caregiver of her aged father (76 years old) who had an accident a few years prior and lost mobility in his right leg. Her presenting complaints included feelings of depression, guilt, anger and resentment at having to be the primary caregiver to her son and father. She was desperately seeking for a special school where she could place her child, hopefully remarry, find another caregiver for her father and ‘move on’ with her life.

## Common themes

Even though these women sought therapy at different times of their ‘journey’ as informal caregivers, there were several common experiences they shared about their role as caregivers of elderly and disabled parents. Some of the presenting issues had to do with the client's own internal processes (e.g. feelings of depression, anger and guilt; reappraisal of motive for role as caregiver), and others had to do with their relationships with significant others, including the care recipient and their own siblings, which affected both their quality of life as well as their caregiving.

Though differing in background and experiences, the dominant emotions expressed were generally depressive and negative, suggestive of poor well-being and satisfaction with life. Interestingly, what these clients reported is also common among other informal caregivers.^2^ For coherence, the common intrapersonal dynamics observed among the clients will be presented, followed by the interpersonal issues and challenges.

## Intrapersonal processes

### Reappraisal of motive and role as caregiver

In the initial stages of therapy, all of the clients appeared to be struggling with coming to terms with their role as caregivers. Likewise, all felt compelled by life circumstances to be informal caregivers to their elderly and disabled parents. Amy (case A) was the only daughter resident in Ghana since her other siblings were all outside the country. Evelyn (case B) seemed to have taken up the role in conformity to the expected cultural norm or duty for an only daughter, that is, filial piety. Mercy (case C) was thrust into the role because of the divorce from her husband and the need to return to the family home. They struggled to find a more intrinsic ‘reason’ for assuming the role other than what seemed obvious and thereby make the experience ‘meaningful.’ Meaning-making appeared to be a coping strategy for all of them.

Working with these clients, it became apparent that for the experience of caregiving to be meaningful, it is important that caregivers clarify for themselves the motive for assuming the role. Is it out of filial piety? Is it to do what is expected of a dutiful daughter, son, wife or husband? Is it a voluntary desire to assist another human being in need? The answer may be a ‘mixed bag’ - not readily clarified or forthcoming. However, daring to ask these questions is a good start to finding some answers and clarity. For all three women, this was an important aspect of the therapy that helped them attain some peace of mind and acceptance of the challenges of their role as informal caregivers. As Amy (case A) later put it: ‘I didn't realise that this experience will change me in the ways that it has’. Indeed, Wicks (2003:3) affirms that ‘difficult times and situations can offer graced moments in more striking ways than the good times can’. However, before positive reappraisal and appreciation are possible, suffering is sure and can only be embraced with clarity of intention and meaning-making of an experience (Wallhagen & Yamamoto-Mitani 2006).

### Depression

Another common presentation among the clients was depression, which is also common among informal caregivers (Lavela & Ather 2010; Navaie-Waliser et al. 2002; Pinquart & Sörensen 2007), and tends to be higher among those who take on the caregiving role involuntarily (Savage & Bailey 2004). Symptoms of depression include feelings of sadness, loneliness and helplessness. Amy expressed it as ‘watching someone you love gradually dying before your eyes […] and there's very little you can do about it’. For her, observing her mother ‘shrink into a skeleton’ of her once full-bodied self as she battled with diabetes was particularly hard and even more so knowing that there was not much she could do. It engendered a sense of sadness and helplessness that she found difficult to talk about.

Depression also sometimes presents as boredom and anhedonia. Evelyn (case B) found ‘always being home and doing the same things over and over’ both boring and depressing. She described the humdrum of assisting her mother with the activities of daily living as challenging, boring and depressing. The repetitive nature of assisting with daily activities and the accompanying boredom can spur the onset of other symptoms, like physical and mental fatigue, anhedonia, loss of concentration, forgetfulness, irritability, changes in mood and vegetative behaviours. Self-esteem issues are particularly evident among older caregivers (Lavela & Ather 2010; Pinquart & Sörensen 2007) and persons who provide caregiving for a long time (Savage & Bailey 2004).

Depression in the caregiver may not present as major depression, but can be dysthymic in nature. Feeling ‘hemmed in’ and experiencing a loss of pleasure in previously enjoyed activities is characteristic of a mild and chronic form of depression known as dysthymia. In describing her lack of joy and feelings of constraint in what she did, Mercy (case C) expressed her frustration:

[*Y*]ou do things you would rather not do and almost never get to do the things you want to do. It is as though your life has been completely taken over by another.

These conflicting emotions and frustration of unfulfilled personal longings have also been observed in other caregivers (Mwinituo & Mill 2006; Navaie-Waliser et al. 2002).

Fatigue in the caregiver is both physical and psychological (Lavela & Ather 2010; Swanson et al. 1997). While physical exhaustion is associated with the mundane task of providing care (Pinquart & Sörenson 2007), psychological exhaustion is compounded by behavioural problems of the care recipient and the quality of the caregiver-recipient relationship (Juster & Marin 2011; Kasaya, Polgar-Bailey & Takeuchi 2000; Savage & Bailey 2004). Caring for an infirm and visually impaired person with a high level of dependence is particularly tasking, as was the case for both Evelyn (case B) and Amy (case A). Both clients reported a psychological and emotional fatigue that was an ever present ‘sense of responsibility’ that ‘sits on the mind’, even when one is physically away from the care recipient. The mind is constantly preoccupied with concerns or thoughts of the sick so that, away or near, the caregiver is mentally tethered. The extent of emotional drain and burden of care is moderated by age (Lavela & Ather 2010), gender (Larrañaga et al. 2008), self-esteem and knowledge (Aziz, Salama & El-Soud 2012; Okoye & Asa 2011; Savage & Bailey 2004), status as primary or secondary caregiver (Vincent-Onabajo, Ali & Hamzat 2013), and attachment to the care recipient (Ainsworth 1989; Navaie-Waliser et al. 2002). The psychological and emotional fatigue, also referred to as ‘burnout’, sometimes expresses as irritability - also symptomatic of depression. In this state, the quality of care and health of both the caregiver and care recipient are compromised.

### Conflicting emotions of anger and guilt

Feelings of anger and/or guilt are not uncommon among informal caregivers (Galluzzi 1999; Kasaya et al. 2000; Vellone et al. 2011). Both Amy and Evelyn presented symptoms of depression, tinged with anger and guilt. Amy's role as caregiver was somewhat voluntary, but she was angry with her ‘absent’ siblings who criticised instead of appreciating her caregiving sacrifices for their mother. Similarly, Evelyn (case B) and Mercy (case C) resented their role and felt forced into it by life's circumstances. Similar resentment or anger has been found among other informal caregivers who assumed their role involuntarily (Lavela & Ather 2010; Ogunlana et al. 2014; Savage & Bailey 2004).

The anger observed in these women, though differently motivated, was mixed with guilt, and the presentation of guilt was different for each of them. Amy and Mercy felt guilty over their own feelings of anger and resentment about their role. Additionally, whenever Mercy left her son or father in another's care and went away for a few days, she reported feeling guilty. Evelyn's guilt was an offshoot of her own awareness of self-directed anger because she felt she was not conforming to the cultural expectation of being a ‘good daughter’. At other times, she reported feeling guilty and uncertain about whether she is ‘doing enough’ for her mother; thus her feelings were often conflicted. Feelings reported by all clients were complex and not easily named or sorted.

Validating and affirming that such feelings were not unusual or uncommon in caregivers (Aziz et al. 2012; Durant & Christian 2006; Goodhead & McDonald 2007; Savage & Bailey 2004; Vellone et al. 2011) helped to normalise their experience and reassured them. They found such validation supportive and therapeutic. Disclosure, appropriately timed and used by the author as their therapist, was also reportedly helpful to them. Amy was particularly grateful during one session and confided, ‘I thought something was wrong with me […] that [I] am a bad person for having such thoughts and feelings […] I feel lighter because at last someone understands’. The author's ability to assist these clients was not only attributable to clinical skills, but also to own experience of being an informal caregiver to an elderly and visually impaired mother. A personal and intimate understanding of them was something they found comforting.

## Interpersonal processes

Aside from their own struggles, clients also reported external interpersonal impacts from significant others that affected their lives as informal caregivers. The most commonly reported experiences centred on their own family members and sometimes their relationship with the care recipient.

There were reports of being ‘taken for granted’ and feeling unappreciated, which is another challenge some informal caregivers encounter (Mwinituo & Mill 2006; Navaie-Waliser et al. 2002; Swanson et al. 1997). Consistent with previous findings, both Amy and Evelyn talked about how other family members, and sometimes the care recipient, failed to understand or appreciate the daily sacrifices they underwent because of their role as caregivers. Amy was painfully aware of how siblings would call and criticise or ‘give instructions’ on how she should take ‘better care’ of their mother, but appeared unwilling to visit and do same themselves. She explained how these criticisms and complaints, instead of the expected affirmation and support, left her feeling very lonely and frustrated.

Lack of support adds to the burden of care and feelings of depression and loneliness, which are common among informal caregivers (Goodhead & McDonald 2007; Lavela & Ather 2010; Pinquart & Sörensen 2007; Savage & Bailey 2004). Support, especially from significant others, can be a great source of strength and solace for both the care recipient and the caregiver. With support, the task of providing care, though difficult, is less burdensome (Lutgendorf & Laudenslager 2009). The lack of appreciation and feelings of being taken for granted are more painful when the care recipient seems to appreciate and/or value family members other than the caregiver. Both Amy and Evelyn talked at length about how their parents appeared to show greater appreciation, affection and tolerance for the behaviour of their other siblings when they called or visited even for a short time.

Informal caregivers may also face the challenge of how to deal with challenging family responses to their role. Instead of support, some family members may misconstrue and misinterpret the caregiver's behaviours towards the care recipient. Decisions by the caregiver, however minor and inconsequential, may be seen by siblings or extended family members as ill-intentioned attempts to usurp their positions in the family or to curry favour with the care recipient for personal gain. Amy recounted many such painful experiences with absent older siblings who failed to understand her decisions about their mother's well-being. In the Ghanaian cultural context, such familial conflicts with consequent feelings of alienation can be very challenging. While noting that such misunderstandings may arise from ignorance about the caregiver's daily realities, it may well be projections of siblings’ own issues and frustrations with themselves about their inability or unwillingness to do for a loved one what the caregiver has been doing. Looking at it this way helped Amy cope.

## Therapy outcome as recommendations for caregivers’ self-care

By the time of termination, the following were observed and reported by clients as factors contributing to their progress. These are noted as indicators of a positive therapy outcome and are therefore recommended for the self-care of informal caregivers, as they may be applicable to persons in similar caregiving situations.

### Awareness

Awareness is important to change. Caregivers need to be aware of how the experience of caring for another is impacting them. Mindful awareness of needs and feelings, as well as the validation of these, enables a person to name your feelings and desires without judgement. Such gentle awareness provides the basis for acceptance and a conscious transformation of the experience. Once the clients became more self-aware as therapy progressed, their ability to cope improved. This is a first step to self-care.

### Meaning-making and positive reappraisal

The author has learnt from own experience of being an informal caregiver, and from the stories of these women ,that sometimes the most precious gifts in life come wrapped in tattered covers. To make a positive reappraisal means to look for the ‘buried treasures’ in the ‘debris’ of the challenge of caregiving. At the time of the experience, it may be considered everything but gifts. Providing care for another does not only put one in touch with your own humanness and vulnerabilities, but it can also be an uplifting experience - the kind that comes from knowing that you are making a difference in another's life (Kleinman 2009).

The experiences of these women revealed that, even though all of them believed that it was their responsibility to care for their elderly and disabled parents, some initially reported a tinge of resentment, partly attributed to lack of support and partly to frustrations associated with the role of caregiving - being extrinsically motivated added to the burden of care. As a means of self-care, positive reappraisal and meaning-making provides a nourishing oasis for a caregiver and shields from burnout because the ‘burden’ of care is seen from a more positive, and therefore more accepting, perspective. The ‘new way of seeing’ and the meaning one makes of the experience helps ameliorate and transform the difficulties. The caregiver might even be pleasantly surprised to find that there are a lot of positives in that difficult, and often underappreciated, role. Evelyn, for example, admitted that it took a while before she ‘saw and admired’ her mother's courage in living with visual impairment. She explained:

I watch with admiration how she takes all her medications without much complaint […] sits in the living room, day in and day out, doing the same things over and over […] and with a sense of humour and gentle appreciation for small gestures of kindness done her […] she has her down days, but I often wonder if I can live as gracefully when I am her age and so handicapped.

Once in this space of positivity, though the difficulties of providing care remained the same, she was better able to respond and cope.

### Support

Clients who had fewer support networks seemed more stressed. It appears that the fastest road to burnout involves taking care of others without paying attention to relaxation and caring for one's own needs. Having a good social network of trusted persons for emotional reliance and support is critical to a caregiver's well-being (Galluzzi 1999; Lutgendorf & Laudenslager 2009). Sharing joys and frustrations with trusted and supportive friends or family can be very uplifting. Persons who make one feel safe, ‘heard’, not judged, and with whom one is free to name one's feelings and fears offer the truest gift of presence that heals. Given the psychological burden of caregiving, it is essential that caregivers monitor their physical health and psychological energy to ensure the provision of quality service.

### Time off

Change of environment has a renewing effect. Time away from the care recipient must be done regularly without feelings of guilt. Respite for caregiver is crucial for sustained energy; time away from the home is therapeutic (Aziz et al. 2012). All the women longed for personal time and sometimes felt guilty and ‘selfish’ for taking time for themselves. However, time away from the care recipient renewed and improved the quality of care they could provide.

### Exercise

Moderate exercise was therapeutic as well. Caregiving is a chronic stressor, which can be reduced through exercise to elevate mood and improve energy level. When Mercy shared that ‘my morning walks clear my mind’, the author could not agree more. It is important to include some form of exercise in one's daily routine as a caregiver.

### Forgiveness

This was something all clients had to work through and resolve. Since the role of caregiving is fraught with challenges that task both the caregiver and care recipient, the art of constant forgiveness is part of self-care. It is insightful to consider that the Greek words for forgiveness are translated most clearly as ‘to release or set free’ and to ‘offer a gift of grace’. Therefore, to engage in the process of forgiveness is a *gift* in itself that is given because there is a certain powerlessness - an unableness - that accompanies deep hurt. To be able to forgive, it is important to be able to name one's pain in order to sever the pain nerve linking the soul to the injury, to cease to define oneself as a victim, and to free oneself from a cluster of old thoughts, ruminations, broodings and grudges. All clients had to deal with some aspect of forgiving – themselves, the care recipient, or significant others with whom they have meaningful relational ties. And once they worked through it, they seemed more peaceful - for when we discover the art of forgiveness, we find new perspective. John Patton (1985), a pastoral counsellor, puts it succinctly:

…forgiveness is not something we do; it is something we discover. I am able to forgive when I discover that I am more like those that hurt me than unlike them; more similar than different. (p. 85)

### Journaling

Since all three clients were literate, they were introduced to something the author herself had found helpful in her self-care as an informal caregiver - journaling. This is the art of writing down thoughts uncensored and unedited. It provides a safe outlet for feelings and thoughts. Besides trusted friends or family who provide support, journaling is helpful because not everything can be shared with even the most supportive of friends or family - either for fear of being misunderstood or because the emotions and thoughts are jumbled up beyond articulation. When this happens, journaling - simply writing for oneself - is great catharsis! The type of journaling that was recommended to these clients, which they found helpful, is called gratitude journaling. Each day, one recalls and writes a single thing for which you are grateful. Caregiving is prone to depression and negativity. When the mind is thus susceptible, it is helpful to focus on the small positive things for which one can be thankful and write about those. It helps train the mind to focus on the positives that otherwise go unnoticed or are taken for granted. Like toning a muscle with practice, the mind gradually focuses on the positive things instead of the negatives. This way, mental strength is fortified.

### Trust in a higher power

Finally, and most importantly, trusting in a power bigger than their own problems and relying on same for assistance is important to self-care. Beliefs are important to coping. The Ghanaian culture is very spiritually oriented. All women alluded to beliefs in a supernatural power as their source of solace and strength. Mercy shared at length that:

…in caring for my mentally challenged son and my father, my greatest and most treasured lesson has been to trust God more […] and I found God to be faithful […] I know from my experience that God always comes through and that the Almighty can be trusted to give strength and support, sometimes from the most unexpected places! […] many of my experiences have become ‘eye openers’ to a greater appreciation of the beautiful complexities of God's world. My conclusions are: it is better to trust in God than to trust in human beings […] because God's grace is sufficient for us […] and we can do all things because he strengthens us!

Again, this could not have been said better!

## Conclusion

In the present day Ghana, care for the elderly, who may also be disabled, is problematic - partly because of ‘empty homesteads’ and constrained availability of family caregivers, and partly due to the time-pressured demands of the modern Ghanaian lifestyle that hardly leaves any time for self or others. In addition, the cultural values of respect and care for the elderly are waning, being eroded by all kinds of external influences - a situation further compounded by lack of well-established professional care centres. Therefore, informal caregiving is predominantly practiced. This too is fraught with challenges, including lack of adequate financial and emotional support from significant others, as corroborated by the shared experiences of the informal caregivers reported in this study.

The experiences of the three women offer some germinal insights into the beauty and complexities of informal caregiving for elderly persons with disability within the Ghanaian context. The goal of this work was to bring to the fore some of the challenges and issues related to the provision of care for the elderly in the present day Ghanaian society for context-specific response and intervention.

Notwithstanding the potential limitation of a small sample size, the importance of support and self-care for the informal caregiver in this cultural context cannot be underestimated. Furthermore, the experiences of these women suggest that there is a niche for professional care facilities for the elderly, who are disabled by age-related complications, in the country. Informal caregiving, by family members or hired hands, is no longer a sufficient response to the social needs of the times as far as elder care is concerned, especially in the cities. Establishment of professional elder care centres by local government and non-governmental agencies would not only serve a social need, but also be a step in the right direction towards the provision of proper and adequate care for the elderly disabled in Ghana.

## Footnotes

1 Real names are not used.

2 Cf. Akintola 2010; Aziz et al. 2012; Gibson & Houser 2007; Juster & Marin 2011; Lahaie, Earle & Heymann 2013; Lavela & Ather 2010; McClelland 2013; Mwinituo & Mill 2006; Navaie-Waliser et al. 2002; NurFatihah et al. 2013; Okoye & Asa 2011; Reinhard et al. 2008; Rizk, Pizur-Barnekow & Darragh 2014; Rombough, Howse & Bartfay 2006; Savage & Bailey 2004; Ziemba 2002.
